# Burnout syndrome in resident physicians: A study after the third COVID-19 wave in two tertiary hospitals of southeastern Brazil

**DOI:** 10.1371/journal.pone.0321443

**Published:** 2025-04-07

**Authors:** Aline Camile Yehia, Janaina Moreira, Melissa Orlandin Premaor

**Affiliations:** 1 Pós-Graduação em Ciências Aplicadas à Saúde do Adulto, Federal University of Minas Gerais, Belo Horizonte, Brazil; 2 Department of Pediatrics, Medical School, Federal University of Minas Gerais, Belo Horizonte, Brazil; 3 Department of Clinical Medicine, Medical School, Federal University of Minas Gerais, Belo Horizonte, Brazil; George Mason University, UNITED STATES OF AMERICA

## Abstract

**Background:**

In recent years, there has been an increase in the concern for the mental health of resident physicians. The COVID-19 pandemic may have further contributed negatively to the mental health of this population.

**Objectives:**

We aimed to evaluate the probability of Burnout Syndrome in resident physicians involved in COVID-19 care services during the Pandemic and possible factors associated with it.

**Methods:**

A cross-sectional study was conducted in two tertiary hospitals in Belo Horizonte, Brazil, between the 5th of June and the 14th of September 2022. A survey including the instruments Oldenburg Burnout Inventory (OLBI), Depression, Anxiety and Stress Scale (DASS-21), Patient Health Questionnaire (PHQ-9), and the Brief Resilient Coping Scale (BRCS) was applied.

**Results:**

From the 181 resident physicians invited to participate, 104 agreed. The mean age (SD) was 29.9 (3.3) years; 56.7% were female, and 67.3% were from a clinical residency program. The score of the OLBI was high. In the multivariate analysis, being single, using psychiatric medications, and taking direct care of COVID-19 were associated with increases in the OLBI scale scores. The frequencies of probable depression and anxiety assessed by DASS-21 were 15.3% and 5.7%, respectively. Notwithstanding, the frequency of probable stress was 61.5%. Depressive symptoms, as evaluated by the PHQ-9 questionnaire, were highly prevalent at 61.5%. Further, 29% of the resident physicians interviewed in our study had probable low resilience according to the BRCS score.

**Conclusion:**

The frequency of Burnout, depression, and stress found in our study appears to be relevant in the resident physicians.

## Introduction

The mental health of physicians and health professionals has been an increased concern of medical educators, the scientific community, and administrators. Burnout Syndrome has been increasingly described in this population [1,2]. This Syndrome is characterized by emotional exhaustion, depersonalization (including a pessimistic and cynical attitude towards work), and feelings of diminished fulfillment at work [1,2]. Moreover, the drivers of this Syndrome are rooted in healthcare organizations and systems, as they include excessive workloads, inefficient work processes, administrative burdens, work-home conflicts, lack of information or control for physicians regarding issues that affect their professional lives, organizational problems, lack of support structures, and a failed leadership culture [1–3].

Although the risk factors for Burnout Syndrome are wide-ranging and exist at all stages of medical practice, medical residents seem particularly vulnerable because of long working hours, low autonomy, and varying levels of social support [2,3]. Prevalence rates of Burnout Syndrome close to or greater than 50% have been described in studies that included resident physicians in the United States [2,3]. However, the prevalence of Burnout Syndrome in these medical residents has yet to be defined. Further, it varies according to the study methodology, subpopulation studied, medical specialty, and study location [3–5].

The context originating from the COVID-19 pandemic may have negatively affected the mental health of health workers and resident physicians. The disease outbreak brought about strong psychological pressure for professionals in direct contact with patients affected by COVID-19 [6,7]. Furthermore, resident physicians had to adapt to the changes in patient care and management policies. Sometimes, they had to exercise leadership roles unprepared for their learning period [8]. Factors such as the growing number of cases of COVID-19, the inexperience of health professionals, the lack of vital resources, the excessive workload, and the inability to contain the spread of the disease might have contributed to the mental suffering in this population [9].

In addition to Burnout Syndrome, resident physicians may experience mental distress in other forms. Symptoms such as depression and anxiety are often linked to Burnout and have been documented in various studies [10–13]. It is important to describe the symptoms related to the psychological suffering of resident physicians to enhance our understanding of Burnout Syndrome.

Even though the COVID-19 pandemic appears to have aggravated the Burnout Syndrome and increased its prevalence, its actual effect on this population is not well established. This study aimed to evaluate the prevalence of Burnout Syndrome among resident physicians who worked during the COVID pandemic. Resident physicians were assessed after the third wave of the Pandemic in Brazil. The objective of this study was also to identify possible factors associated with the occurrence of Burnout Syndrome in this specific population.

## Methods

A cross-sectional study recruited resident physicians assigned to care for patients with suspected or diagnosed COVID-19 between the 5th of June and 16th of September of 2022 in two tertiary hospitals (Hospital Luxemburgo and Hospital Risoleta Tolentino Neves) in the city of Belo Horizonte, Brazil, was carried out. The recruitment occurred at the same time in both study sites. All resident physicians scheduled to care for COVID-19 patients were invited to participate via e-mail and WhatsApp. The resident physicians who agreed to participate received a link with the informed consent and the survey.

The study followed the Helsinki Convention and Resolution 466/12, which regulates ethics in scientific research in Brazil. All participants signed an electronic informed consent form. The survey was only availiable to the participants after the signature of the consent form. All the participants’ data were anonymized and kept confidential in the survey. The study was approved by the Ethics Committee of the Federal University of Minas Gerais (CAAE 51478621.6.0000.5149) and the Ethics Committee of the Hospital Luxemburgo/Fundação Mário Penna/Associação Mário Penna (CAAE 51478621.6.3001.5121).

Resident physicians who agreed to participate completed the study questionnaires on Google Forms. All fields were marked as mandatory, so the participant could only advance after answering all the questions. Information regarding the following demographic variables was collected: age, gender, marital status, medical specialty, year of residence, years after graduation, year of entry into the residency, who the participant lives with, use of medication (yes or no), interruption of classes, continuity of in-person classes, comorbidities (yes or no), the vaccine for COVID-19.

The following questionnaires were applied to assess the mental health of resident physicians: Oldenburg Burnout Inventory (OLBI) which evaluated the probability of the presence of Burnout Syndrome [14,15] the Depression, Anxiety and Stress Scale - 21 Items (DASS-21) [16] was used as a screening tool for depression, anxiety, and stress; the Patient Health Questionnaire depression module (PHQ-9) was used as depression screening [17,18]; the Brief Resilient Coping Scale (BRCS) was used to estimate the degree of resilience [19,20]; and the visual analog scale (VAS) was used to measure the level of suffering at work [21,22]. The question used in the VAS assessment was: “Between 1 and 10, where 1 means no suffering and ten means maximum suffering, how do you rate your suffering caused by the pandemic?”. All these scales were previously adapted and validated for use in the Brazilian population [14–17,19,20,22]. Furthermore, the OLBI inventory validated for Brazil and used in our study included 14 questions instead of the original 16. Two questions were excluded from the Brazilian version during the validation process because they have low discriminatory potential in our country [14,15].

Because there is no cut-off point for the OLBI inventory associated with adverse outcomes over time in Brazil, we used the cut-off associated with Burnout Syndrome diagnosis in the psychiatric consultation as suggested by Delgadillo et al. [23]. They found that one standard deviation (SD) above the sample mean is associated with Burnout Syndrome Diagnosis. To calculate the mean values and SD, we have used the study of Pinho et al. [24], conducted on Brazilian resident physicians and has a methodology similar to ours. Therefore, our study’s cut-off point for probable Burnout Syndrome was >  3.91 (1 SD) [23,24]. urther, we also performed a sensitivity analysis using two standard deviations. We chose two standard deviations because they are adopted for statistical normality. For this analysis, the cut-off was 4.65 (2 SD).

The symptoms of exhaustion and detachment were considered present according to the normality criterion; therefore, we used 2 SD for this purpose. When the study participants had scores greater than 4.53 and 5.09, they were considered positive for the detachment and exhaustion domains of the OLBI inventory, respectively [24].

Probable low resilience was considered present when BRCS scale scores were less than 13 [25]. Probable depression assessed by the PHQ-9 scale was considered present when scores on this scale were equal to or greater than 9 [17]. The DASS scale was used as an instrument to assess depression, anxiety, and stress. We interpreted this scale based on the tripartite model of anxiety and depression, which encompasses the specific symptoms of depression and anxiety in separate factors and groups them into a tripartite model. This model proposes that affective disorder (and its subtypes) is a continuum between depression, anxiety, and stress [16]. According to this model, stress is the most severe form of the disease. We chose this form of interpretation a priori because we hypothesized that the symptoms of stress could be more associated with Burnout Syndrome than the others. Probable anxiety, probable depression, and probable stress were considered present when the scores obtained on the DASS scale were greater than 7, 9, and 14, respectively [16].

### Statistical analysis

Population characteristics were described as mean [standard deviation (SD)], median [interquartile range (IQR)], and frequency [absolute number (n/n total) or percentage]. Data were tested for normality by inspecting the histogram and assessing Skewness and Kurtosis. Probably Burnout Syndrome was considered present when the OLBI inventory score was greater than 3.91 (1 SD). Further, a sensitivity analysis was performed to evaluate the probability of Burnout Syndrome according to the normal distribution. In the latter, probable Burnout Syndrome was considered present when the OLBI inventory score was greater than 2 SD (4.65).

Due to all resident physicians being considered to have probable Burnout Syndrome using the above cut-offs, we also explored the OLBI inventory quartiles as a continuous variable. Our rationale was that the physicians in the upper quartiles could be different from those in the lower quartiles, indicating greater severity in the upper quartiles.

Anova and Chi-square tests were used to evaluate the association of the variables with the OLBI quartiles. Univariate analysis was performed using simple linear regression, considering the OLBI inventory scores as the dependent variable. Multiple linear regression analysis was used to assess possible factors associated with higher scores on the OLBI inventory. The final multiple linear regression model was chosen using the backward technique, with p-values of 0.10 for entry and retention in the model. Analyzes were performed using SPSS 18.0 (ARMONK, USA). P-values < 0.05 were considered significant. The data is available at Figshare [26].

## Results

Of the 181 resident physicians working from June to September 2022, scheduled to care for patients with suspected or diagnosed COVID-19 in the two hospitals studied, 104 agreed to participate in the survey. Participants’ mean (SD) age was 29.9 (range 25–40) years. 56.7% were female, 67.3% were enrolled in clinical residency programs, 29.8% in surgery residency programs, and 2.9% in anesthesiology residency programs. The characteristics of the resident physicians included in the study according to the quartiles of the OLBI scale are shown in Table 1.

**Table 1:** Characteristics of the studied resident physicians according to the quartiles of the *Oldenburg Burnout Inventory* (OLBI).

Characteristic	1^st^ quartile * n = 24	2^nd^ quartile* n = 22	3^rd^ quartile* n = 25	4^th^ Quartile * n = 21	p-value
Age (year)	30.4 (3.5)	30.2 (3.7)	30.0 (3.1)	29.0 (3.2)	0.455
Gender					
Female	10	14	16	12	0.365
Marital status					
Single	13	15	19	17	0.214
Specialty of residence					
Clinical	15	11	17	17	0.110
First year of Residency	6	5	6	4	0.458
Year after graduation	3.6 (2.4)	3.9 (3.0)	3.9 (2.4)	2.8 (1.8)	0.381
Admission to residency after 2020	9	8	11	15	0.072
Lives alone	5	6	8	8	0.627
Lives with a person who works in the health area	10	7	12	8	0.721
Vaccinated against COVID-19	24	22	25	21	–
Comorbidities	5	1	4	4	0.427
Use of medication	3	5	5	10	0.044
Use of psychiatry medication	3	5	10	12	0.008
Direct response to COVID-19	22	22	25	21	0.122
Had classes interrupted					
Fully	8	7	11	4	0.081
Partially	7	10	9	15	
Proportion of in-person classes					
100%	0	0	2	1	0.522
>= 50%	1	2	2	1	
< 50%	10	9	4	6	
0%	13	11	17	3	

Data are described as mean (SD) or as n.

* The OLBI escores for the quartiles were:

1^st^ quartile: 17 to 29.24.

2^nd^ quartile: 29.25 to 43.99.

3^rd^ quartile: 44 to 48.99.

4^th^quartile: 49–59.

The frequency of probable Burnout Syndrome assessed by the OLBI scale was 100% when we consider both cut-off points 3.91 (1 SD) and 4.65 (2 SD) described in the study by Pinho et al. [24] (Table 2). The mean scores of the OLBI scale found in the resident physicians included in our study were 43.3 (SD 8.2), 21.7 (SD 4.1), and 21.6 (SD 4.9) for the total score and the detachment and exhaustion domains, respectively. These subjects also had high scores on the DASS scale [median 19 (IQR 10.3; 28.5)], PHQ-9 scale [median 10.6 (IQR 6.0;14.8)] and the distress score assessed by the VAS scale [6.4 (SD 1.7)]. The average score on the BRS scale was 14.0 (SD 3.2). The frequencies of Burnout Syndrome, depression, anxiety, stress, and low resilience assessed by the scales applied in our study per the OLBI quartiles are described in Table 2. Twenty-nine percent (29%) of the resident physicians interviewed in our study had probable low resilient coping according to the BRCS score. Further, the frequencies of probable depression, anxiety, and stress assessed by the DASS-21 questionnaire were 15.3%, 5.7%, and 61.5%, respectively. Also, the presumptive diagnosis of depression by the PHQ-9 questionnaire had a high prevalence, 61.5%.

**Table 2:** The frequency of symptoms suggestive of depression, anxiety, stress, low resilience, and Burnout Syndrome accessed by the applied scales in the studied resident physicians.

Instrument	1^st^ quartile * n = 24	2^nd^ quartile^*^ n = 22	3^rd^ quartile^*^ n = 25	4^th^ Quartile ^*^ n = 21	p-value
DASS					
Probable Anxiety	2	0	3	0	<0.0001
Probable Depression	5	9	3	1	
Probable Stress	7	8	18	29	
DASS score	9.5 (IQR 3.3;16.5)	12 (IQR 10;20.8)	18 (IQR 12.5;26.5)	32 (IQR 23.0;40.5)	<0.0001
PHQ-9					
Probable Depression	8	11	17	19	0.001
BRCS					
Probable Low Resilience	5	9	6	9	0.254
VAS	6.0 (IQR 4;7)	6.5 (IQR 4.8;7.3)	7.0 (IQR 5.5;8.0)	8.0 (IQR 7;8.5)	<0.0001

Data are described as n or median (Interquartile interval - IQR).

Probable low resilience was considered when the BRCS scale scores were less than 13.

Probable depression assessed by the PHQ-9 scale was considered present when scores on this scale were equal to or greater than 9.

Probable anxiety. probable depression. and probable stress were considered present when the scores obtained on the DASS scale were greater than 7. 9. and 14. respectively.

* The OLBI escores for the quartiles were:

1^st^ quartile: 17 to 29.24.

2^nd^ quartile: 29.25 to 43.99.

3^rd^ quartile: 44 to 48.99.

4^th^quartile: 49–59.

In the univariate analysis, being single, doing the residency in a clinical area, the year of entry into the residency, use of medication, use of psychiatric medications, performing direct care for COVID-19, and having the classes of the residency interrupted were directly associated with an increase in the score of the OLBI scale (Table 3). On the other hand, a longer period in residence and the BRCS scale scores were inversely associated with the OLBI scale score (Table 3). In the multivariate analysis, being single, psychiatric medication use and direct care for COVID-19 remained positively associated with an increase in the OLBI scale score in the final linear regression model. These findings are shown in Table 4.

**Table 3:** Univariate linear analysis models using OLBI scale scores as the dependent variable.

	B (SE)	Beta	p-valor
Age (year)	-0.43 (0.2)	-0.17	0.086
Female Gender	-2.90 (1.6)	-0.18	0.074
Marital status – single	4.20 (1.7)	0.23	0.018
Specialty of residence – Clinical*	4.0 (1.8)	0.22	0.025
Year of residency – (last year)	-0.64 (0.7)	-0.22	0.028
Admission to residency	1.50 (0.7)	0.21	0.031
Live alone	2.13 (1.7)	0.12	0.227
Lives with a person who works in the health area	1.41 (1.7)	0.08	0.398
Comorbidity	0.40 (2.1)	0.02	0.857
Had COVID-19	-0.43 (2.3)	-0.22	0.853
Use of medication	4.0 (1.8)	0.22	0.025
Use of psychiatric medication	5.71 (1.6)	0.33	0.001
Direct response to COVID-19	14.46 (4.6)	0.30	0.002
Had classes interrupted	2.17 (1.0)	0.21	0.030
Proportion of in-person classes	-0.02 (1.0)	-0.0	0.985
BRCS scores	-0.70 (0.2)	-0.27	0.005

*when compared with non-clinical.

**Table 4:** Multiple linear regression model using OLBI scale scores as dependent variable.

	B (SE)	Beta	p-valor
Marital status – single	3.40 (1.6)	0.19	0.042
Specialty of residence – Clinical*	3.10 (1.6)	0.17	0.061
Use of psychiatric medication	5.01 (1.6)	0.28	0.002
Aten Direct response to COVID-19	11.46 (4.4)	0.23	0.011

Final multiple linear regression model using the backward technique. The following variables were included in the initial model: marital status; year of entry into the residency; type of residency (clinical or surgical); length of residency; use of psychiatric medications; direct response to COVID-19; interruption of residency classes; and BRCS scale score.

*when compared with non-clinical.

OLBI scale scores were positively associated with DASS, PHQ-9, and VAS scores in the univariate linear regression analysis (p-values < 0.001, < 0.001, and 0.005, respectively. When we evaluated the association between positive screening of anxiety, depression, and stress generated by the DASS scale, stress scores were positively associated with higher OLBI scale scores [B 4.0 (SE 0.6), Beta 0.56, p-value < 0.0001]. Likewise, the positive screening of depression using the PHQ-9 scale was associated with higher scores on the OLBI scale [B 7.1 (SE 1.5), Beta 0.42, p-value < 0.0001].

## Discussion

In our study, evaluated individuals had a high probability of experiencing burnout syndrome. All resident physicians included in the study exhibited Burnout Syndrome symptoms in both dimensions of the OLBI inventory: exhaustion and distance from work. It should be noted that our study was conducted after the three waves of the COVID-19 pandemic in Brazil.

Before the COVID-19 Pandemic, the prevalence of Burnout Syndrome reported in resident physicians ranged from 23.0% to 100% depending on the study methodology, study design, study location, and type of residence [4,5,27]. Studies carried out during the Pandemic have also described a wide variation in the frequency of this Syndrome [11,28–30]. Furthermore, the results of the studies regarding the increase in prevalence after the Pandemic are also unclear [11,28,29]. A systematic review that assessed the frequency of probable Burnout Syndrome estimated during the Pandemic estimated by a previously validated questionnaire in resident physicians found a prevalence of probable burnout syndrome of 40% (26–57%). It is important to mention that in this systematic review, only two studies included in this systematic review were conducted in South America (Brazil and Argentina). [31]. Further, a study conducted in China during the Pandemic that used the snoll-bol methodology and assessed Burnout Syndrome through a specific questionnaire found a frequency of 43% of self-reported burnout syndrome [32].

Studies conducted with resident physicians worldwide in 2022 also describe different frequencies of Burnout Syndrome. A study conducted in Pakistan in March 2022, which used the MBI, found a prevalence of Burnout Syndrome of 50.7% [33]. In Israel, a survey carried out between March and May 2022, which used its own and a previously validated questionnaire, described that 68% of resident physicians feel frequent symptoms of Burnout Syndrome due to a high number of administrative tasks, and 61% feel frequent symptoms of Burnout Syndrome due to the difficulty in balancing between work life and family life [34]. Another study on resident physicians in Thailand between September and October 2022 assessed the probability of Burnout Syndrome with the MBI, describing a prevalence of Burnout Syndrome of 46.3% [35]. Moreover, a study by the University of Alberta between September and November 2022 assessed the probability of Burnout through the MBI via Google Forms. In this study, the survey had a response rate of 48%, and the prevalence of Burnout Syndrome was 57.9% (95%CI 45.8–69.2%) [36].

More studies are needed to evaluate if the prevalence of Burnout Syndrome decreased after the Pandemic. Li et al. performed a cross-sectional survey of adult and pediatric emergency physicians and trainees in Canada from October to November 2023. They had a response rate of 63.1%. The frequency of probable Burnout Syndrome evaluated by MDI was 44.9% [37].

In our study, the probability of burnout syndrome appears to be higher than that reported in pre-pandemic studies in Brazil [38,39]. A study conducted in northeastern Brazil in 2016 identified a 27.9% frequency of Burnout Syndrome in resident physicians [38]. Another study conducted in 2017 in the city of São Paulo with resident physicians recruited via e-mail found a 63% prevalence of Butnout Synchrome [39]. The response rate in this study was 36.8%. A study conducted with orthopedic residents at the beginning of the Pandemic in 2020 found a prevalence of 69.2% [40]. Furthermore, a study conducted in 2021, also in the northeast, described a prevalence of Burnout Syndrome of 73.1% [41]. In the latter, the response rate was 49.3% [41]. All of the studies mentioned above used the MBI.

On the other hand, a study that compared the frequency of Burnout Syndrome assessed by the MBI in two independent surveys carried out on resident physicians who are members of the Physician Residents National Association (Brazil) found a decrease in the prevalence of this Syndrome after the onset of the COVID-19 pandemic (37.0% versus 26.1%) [11].

Steil et al. evaluated 3071 resident physicians through a convenience sample in April 2020 [42]. The invitation to participate in the study was made through social media, and the response rate was 10% [42]. In this study, the prevalence of Burnout Syndrome assessed by the OLBI was 48.6% [42]. However, we cannot compare our results to this study’s because they used the international cut-off for the OLBI scale. Interestingly, in this study, the frequencies of Burnout Syndrome were different in the different Brazilian regions [43].

Some factors may have contributed to our high prevalence of probable Burnout Syndrome. One might be the high-stress level associated with time exposure to the Pandemic. Our study was carried out after the third wave of COVID-19 [44]. Even though the third wave in Brazil had less mortality than the first wave, there was no reduction in morbidity [44]. On the contrary, the morbidity peaked in the third wave. Furthermore, the national and centralized command of the pandemic confrontation did not happen in Brazil; thus, public administrators took the lead in their territories, which might have contributed to the high prevalence of Burnout Syndrome in the resident physicians during the third wave [44]. Another possible explanation for these findings may be related to the fact that all resident physicians have some involvement with COVID-19 care, regardless of the year of residency [30].

The univariate analysis found that being single, using psychiatric medications, and being involved in direct COVID-19 care were associated with higher OLBI scores. Furthermore, clinical physicians tended to have higher scores on this inventory. The association between the highest scores on the OLBI inventory and adverse outcomes is still not well studied in the literature; however, it is believed that higher scores may be associated with greater disease severity [45,46].

The predictive factors of Burnout Syndrome are conflicting in the literature. However, being single is associated with this Syndrome in some studies [1], but this association was not repeated in others [47]. Nituica et al. found an inverse association between being single/never having been married and emotional exhaustion in US medical residents in the pre-pandemic period [48]. Contrary to our study, Dinibutun et al. described a lower frequency of the Syndrome among physicians who were actively involved in providing care for COVID-19 during the Pandemic compared to physicians who were not actively engaged in providing care [49].

The positive association between Burnout Syndrome and the use of psychiatric medication during the Pandemic, found in our study, is also unclear in the literature. In the work by Prakash et al., for example, resident physicians who used this medication class were excluded [50]. Alkhamees et al., in their meta-analysis on physician Burnout Syndrome during the COVID-19 pandemic, considered the need for further studies in which the presence of psychiatric disorders in Burnout Syndrome was analyzed [10]. The use of psychiatric medication may be related to the high rate of depression that we found. We cannot clarify this issue due to the study’s cross-sectional design. Our question was limited to information on the use or not of psychiatric medication.

The inverse association between resilience and Burnout Syndrome has been widely debated [1,29,45,51]. Almost one third of the resident physicians interviewed in our study had probable low resilient coping according to the BRCS score. Wang et al. described an inverse association between scores on different scales used to assess Burnout Syndrome and resilience in palliative care physicians in Canada [51]. In a study that evaluated the association between resilience (assessed by the BRS scale) and OLBI inventory scores in 130 oncologists after the onset of the COVID-19 pandemic, OLBI inventory scores were lower in those individuals who showed higher resilience in the univariate analysis [45]. In this study, the authors did not perform a multivariate analysis of the factors associated with the OLBI inventory scores [45]. Conversely, Batista et al. found no association between resilience and Burnout Syndrome in primary care physicians [29]. In our study, the BRS scale scores were inversely associated with the OLBI scale scores in the univariate analysis. However, this association did not remain after adjustments in the multivariate regression, suggesting that other factors may contribute to or confound this association.

Several studies have been reporting the association between anxiety, depression, and stress even before the COVID-19 pandemic [11–13], when assessing the influence of the COVID-19 pandemic on depression and anxiety rates, demonstrated an increase in the frequency of depression (46.0% vs. 58.8%), with no change in the frequency of anxiety (56, 5% versus 56.5%) [11]. In our study, we have a high frequency of resident physiscian with stress diagnosed by the DASS scale. As the DASS scale is continuous, and higher values indicate stress, this apparent lower frequency of depression and anxiety suggests a more severe condition with syntoms of depression and stress. Corroborating this hypothesis, the presumptive diagnosis of depression by the PHQ-9 questionnaire had a high prevalence.

In our study, the VAS scale was used as another measure of the suffering that medical residents were experiencing during the Pandemic. The self-reported VAS score showed a direct relationship with the scores on the OLBI inventory, corroborating the psychological distress that was measured by the latter.

There are probably other factors not studied that contributed to the high prevalence of Burnout Syndrome in our study. It is essential to consider that the data collection took place after the third wave of the COVID-19 pandemic in Brazil. Few countries have had three waves of COVID-19 [44]. This fact may have increased the burden, volume, and number of working hours of resident physicians involved in patient care during the Pandemic and, consequently, the increase and severity of Burnout Syndrome. Factors such as a tense work pattern, preceptorship, less leisure time, longer working hours, lack of time off, unsupervised care delivery, wrong choice of specialty, poor learning, and psychological abuse have been described as exacerbating the Burnout Syndrome [12,52].

Our study has some limitations. The first is inherent to the study design, which is cross-sectional. Cross-sectional studies evaluate both the outcomes and study factors at the same time, which does not allow the evaluation of possible causality. Second, the present study relied only on self-report measures, so response bias cannot be ruled out. Nonetheless, the great majority of the studies about burnout rely on self-reported questionnaires. Third, our survey had a response rate of 57,5%. Although we cannot know how the resident physicians who did not respond differ from the ones who did, the rate of response of the other studies is similar to ours. Fourth, the cut-offs used in our study to assess a possible prevalence (1 and 2 standard deviations) have yet to be validated for outcomes such as days lost from work, the appearance of comorbidities, hospital admission, or others. Finally, we cannot rule out that other variables, besides the predictors evaluated by the study, are associated with the development of Burnout Syndrome in our research. Among them are the exposure time needed to face the Pandemic and the workload. The latter was not evaluated because Brazilian legislation requires all resident physicians to work 60 hours a week, and we consider it a sensitive issue to be addressed in a survey.

Burnout syndrome is a significant health issue in medical residency. Therefore, **it is** crucial to consider policies and interventions to alleviate the symptoms and adverse outcomes of Burnout Syndrome in both public health and educational institutions. These measures should aim to prevent and treat Burnout Syndrome. Several strategies have been suggested, including closer contact with the supervisor, access to telemedicine, wellness programs, accommodation resources, daycare, transportation assistance, implementation of flexible schedules, days off, and physical exercise [53–57]. While mindfulness has shown promise in treating Burnout Syndrome, some authors have raised concerns about its time demands [57]. Furthermore, therapy and therapy groups seem to be useful in treating Burnout Syndrome and improving symptoms [55].

In conclusion, the OLBI inventory identified that all resident physicians in our study had some symptoms that could be related to Burnout Syndrome. In addition, being single, on psychiatric medication, and involved in direct care for COVID-19 were factors associated with higher scores on the dimensions of the OLBI inventory. New studies must be carried out to evaluate the long-term outcomes of this Syndrome and its symptoms. Furthermore, strategies for early recognition and mitigation of this Syndrome should be i incorporated by educational institutions.
